# Endoscopic management of hypertensive intraventricular haemorrhage with obstructive hydrocephalus

**DOI:** 10.1186/1471-2377-7-1

**Published:** 2007-01-04

**Authors:** Yad Ram Yadav, Gaurav Mukerji, Ravikiran Shenoy, Abhijeet Basoor, Gaurav Jain, Adam Nelson

**Affiliations:** 1 NSCB Medical College and Hospital, Jabalpur, India; 2 Hammersmith Hospital and Imperial College, London, UK

## Abstract

**Background:**

Intracranial haemorrhage accounts for 30–60 % of all stroke admissions into a hospital, with hypertension being the main risk factor. Presence of intraventricular haematoma is considered a poor prognostic factor due to the resultant obstruction to CSF and the mass effect following the presence of blood resulting in raised intracranial pressure and hydrocephalus. We report the results following endoscopic decompression of obstructive hydrocephalus and evacuation of haematoma in patients with hypertensive intraventricular haemorrhage.

**Methods:**

During a two year period, 25 patients diagnosed as having an intraventricular haemorrhage with obstructive hydrocephalus secondary to hypertension were included in this study. All patients underwent endoscopic evacuation of the haematoma under general anaesthesia. Post operative evaluation was done by CT scan and Glasgow outcome scale.

**Results:**

Of the 25 patients, thalamic haemorrhage was observed in 12 (48%) patients, while, 11 (44%) had a putaminal haematoma. Nine (36%) patients had a GCS of 8 or less pre-operatively. Resolution of hydrocephalus following endoscopic evacuation was observed in 24 (96%) patients. No complications directly related to the surgical technique were encountered in our study. At six months follow-up, a mortality rate of 6.3% and 55.5% was observed in patients with a pre-operative GCS of ≥ 9 and ≤ 8 respectively. Thirteen of the 16 (81.3%) patients with a pre-operative GCS ≥ 9 had good recovery.

**Conclusion:**

Endoscopic technique offers encouraging results in relieving hydrocephalus in hypertensive intraventricular haemorrhage. Final outcome is better in patient with a pre-operative GCS of >9. Future improvements in instrumentation and surgical techniques, with careful case selection may help improve outcome in these patients.

## Background

Non-traumatic intracerebral haemorrhage (ICH), an acute and spontaneous extravasation of blood into the brain parenchyma is the second most common cause of stroke [1] and accounts for 30–60% of all stroke admissions into a hospital [2]. Hypertension results in a two to six fold increase in the risk of intracerebral haemorrhage [3,4]. Thirty day mortality for CT confirmed intracerebral haemorrhage has been reported to be between 34 to 50% [5]. The severity of neurologic presentation [Glasgow Coma Scale (GCS)], age of patient, location of haematoma and presence of intraventricular haematoma are considered predictors of 30 day and 1 year mortality, with the older patient with a lower GCS, infratentorial haematoma and an intraventricular haematoma having a worse prognosis [6]. The expanding haematoma or the occlusion of cerebrospinal fluid (CSF) flow by intraventricular clotting can result in obstructive hydrocephalus and an increase in the intracranial pressure which needs urgent decompression. Methods of reducing the intracranial pressure and relieving the obstruction to flow of CSF include non surgical measures like infusion of mannitol [7], initiators of haemostasis like recombinant factor VIIa [8] to control the bleeding, and surgical techniques like placement of an external ventricular drain to maintain an ICP below 20 mmHg and a minimum cerebral perfusion pressure of 60 mmHg [9]. Other minimally invasive techniques, such as endoscopic evacuation of a haematoma [10,11] and stereotactic CT guided aspiration and thrombolysis [12,13] have also been reported. In this study, we report our results of management of obstructive hydrocephalus secondary to hypertensive intraventricular haemorrhage by endoscopic evacuation of haematoma.

## Methods

Patients with a history of hypertension who developed a ventricular haemorrhage resulting in an obstructive hydrocephalus diagnosed during a two year period from January 2004 to December 2005 were prospectively included into the study. All patients had the diagnosis confirmed by a CT scan pre-operatively. Patients having a large parenchymal haematoma (larger than 60 ml) and those in whom the haematoma extended to the surface were excluded. Patients with an intraventricular haematoma with no evidence of obstructive hydrocephalus were also excluded from the study. A pre-operative assessment was carried out and the neurological status of all eligible patients was recorded using the Glasgow Coma scale. Hypertension was controlled pre-operatively by medical management using antihypertensives and the use of hyper osmotic agents. Patients were operated upon as soon as their blood pressure was controlled and were deemed stable for surgery. The surgery was performed within 24 hours of diagnosis in 9, between 25 – 48 hours in 12, and 49 – 72 hours in 4 patients. All the surgeries were carried out by a senior neurosurgeon (YRY). The study was approved by Central ethical committee of N.S.C.B. Medical College and Hospital, Jabalpur.

### Operative technique

In patients with a putaminal or thalamic haematoma, two frontal burr holes were performed under general anaesthesia. On the side opposite to the haematoma, this burr hole was placed just anterior to the coronal suture that was used to aspirate haematoma from the underlying lateral ventricle and perform a third ventriculostomy. On the side of the haematoma, the burr hole was placed more anteriorly and was used to evacuate haematoma from the lateral ventricle of the corresponding side. Surgery was performed under direct vision using the Rigid Karl Storz 6° scope (Gaab system, Karl Storz GmbH & Co., Tuttlingen, Germany). The side opposite to haematoma was approached first. Sheath was introduced using a stellate and its position in the lateral ventricle confirmed by egress of blood mixed CSF. The sheath was then fixed using a holder and the telescope was introduced. Due to the presence of clots and blood, the initial view inside the lateral ventricle was very poor. The vision gradually improved by continuous irrigation and gentle suction using catheter. The outflow was then closed intermittently for 2–5 seconds to allow visualization of clot and ventricular anatomy. The clot from lateral ventricle body, frontal horn and third ventricle was aspirated and the third ventriculostomy performed. The burr holes were occasionally enlarged to allow little movement in lateral ventricle. This movement in lateral ventricle was only possible in the presence of moderate to large ventriculomegaly; it is not possible or desirable when ventricles were either normal or marginally enlarged. The temporal horn was difficult to access, however, the infant feeding tube could be negotiated into it and clot was carefully aspirated. A rigid Karl Storz 30° scope was sometimes used to visualise the temporal horn and fourth ventricle. Subsequently, the side of hematoma was approached. Haematoma was evacuated by repeated irrigation using lactated Ringer's solution and aspiration through an infant feeding tube (size -6 – 8 Fr), grasping forceps (diameter – 2.1 mm) to crush and remove larger clots and bipolar forceps to cauterize areas of visible bleeding passed through the endoscope's operating channel (3.0 mm). Most of the visible clots from the putamen and thalamus were evacuated. Posterior fossa haematoma was also approached through frontal access, and was evacuated by an infant feeding tube passed through the aqueduct of Sylvius using the technique of gentle irrigation and suction. An external ventricular drain (EVD) was left in place for three days post-operatively in all cases.

Post operative evaluation included clinical assessment using the GCS. CT scan was performed 24 hours after removal of the EVD [Mean (range): 4.86 (4^th ^to 7^th^) post-operative day] and another one at 6 weeks after surgery to confirm the evacuation of haematoma and decompression of hydrocephalus. Patients who did not show satisfactory improvement or developed deterioration had further CT scans carried out. Final outcome was recorded by a Glasgow outcome scale at 6 months following surgery.

## Results

**Figure 1:**
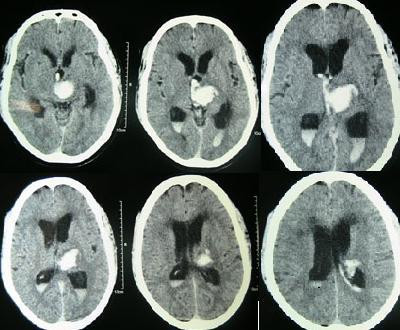
**Pre operative CT scan**. Representative pre-operative CT scan of a patient showing a thalamic haemorrhage with associated hydrocephalus and blood in the third and both lateral ventricles.

**Figure 2:**
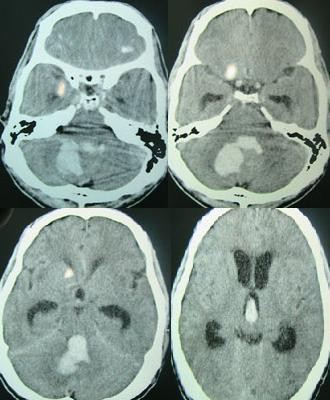
**Pre operative CT scan**. Representative pre-operative CT scan of a patient showing a posterior fossa haemorrhage with associated hydrocephalus and blood in the ventricles.

During the two year period, twenty five patients with a history of hypertension who presented with an intraventricular haematoma were included in the study. The demographic details and the clinical characteristics of study population are summarized in table 1. Thalamus (Fig 1) was the commonest site for the haematoma in 12 (48%) patients followed by Putamen (11, 44%), while two patients had a posterior fossa haematoma (Fig 2) resulting in an intraventricular haemorrhage and obstructive hydrocephalus. The size of the haematoma estimated using a pre-operative CT scan ranged between 30 – 60 ml (Table 1). The pre-operative GCS was 8 or less in nine patients and 9–12 in the remaining sixteen patients.

Patients underwent an endoscopic evacuation of haematoma and decompression of hydrocephalus. A third ventriculostomy was performed in all patients, posterior to the infundibular recess. Duration of surgery ranged from 45 minutes to three hours. There were no complications directly related to the surgical technique.

**Figure 3:**
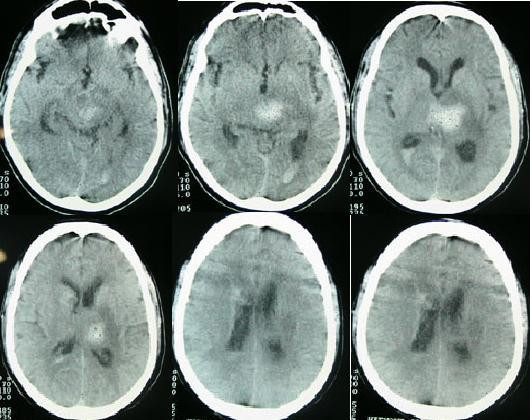
**Post operative CT scan**. Post operative CT scan of the patient depicted in figure 1. Scan done seven days after surgery showing resolving hydrocephalus and the absence of intraventricular blood.

**Figure 4:**
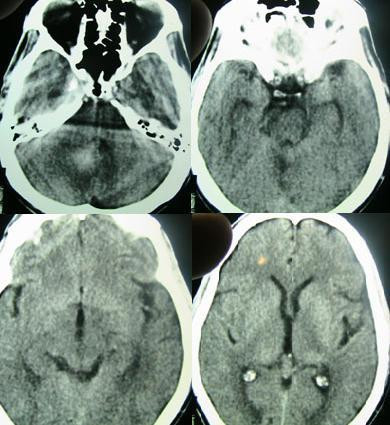
**Post operative CT scan**. Post operative CT scan of the patient depicted in figure 2. Scan done five days after surgery showing resolving hydrocephalus.

The post-operative CT scan (Fig 3 and 4) performed at least 24 hours after removal of EVD confirmed the evacuation of haematoma and relief of hydrocephalus in all cases except one, where the scan demonstrated presence of a communicating hydrocephalus. A repeat CT scan on the 14^th ^post operative day showed the progression of the hydrocephalus, although his neurological status (GCS 13/15) did not deteriorate. This patient improved following a lumbar-peritoneal shunt.

At six month follow-up, 13 of the 16 (81.3%) patients with a pre-operative GCS of 9 and above had a good recovery (Table 2). One patient was left with moderate disability and one had severe disability. One (6.3%) patient from this group died during the 6 month follow-up. Among the patients who had a poor pre-operative GCS (≤8), five (55.5%) patients died while two each were left with moderate and severe disability. None of our patients developed the 'vegetative state'.

## Discussion

Intraventricular haemorrhage (IVH) secondary to hypertensive intracerebral haemorrhage represents a clinicopathologic entity with a dismal prognosis, which is worse when associated with hydrocephalus [14]. A raised intracerebral pressure or herniation secondary to brain tissue injury and swelling is the primary cause of neurological deterioration after the first day [15]. The obstruction to the normal flow of cerebrospinal fluid combined with the mass effect caused by the satellite haematoma within the ventricles and an increase in the intracranial pressure can cause further deterioration. Hence the goal here should be to evacuate the intraventricular haematoma, reverse the ventricular dilatation and restoration of normal intra cerebral pressure [14].

The STICH trial failed to demonstrate that evacuation of haematoma within 72 hours results in a better outcome compared to medical management alone [16], however, this may not be applicable in all cases. Other studies have shown a favourable outcome using neuroendoscopic management of intraventricular haemorrhage [10,14]. In our series, 81.3% of patients with an initial GCS of 9 or more had a good recovery. The final outcome depended mainly on the initial GCS, with just over half (55.6%) of patients with an initial GCS of 8 or less having died within 6 months, with the remaining left with moderate to severe disability. Our results are in agreement with previous studies, which report the main predictive parameter to be the severity of neurologic presentation [17].

Majority of our patients had thalamic or putaminal haematoma. The use of endoscopic approach in such patients has been previously described [11]. Previous studies have reported poor results with endoscopic removal of deep intra parenchymal bleed as these clots are dense and hard when compared to soft fragile ventricular clots [18]. For this reason, patients with a large amount of blood (>60 ml) and those where the bleeding extended to the surface (intra parenchymal bleed) were offered microsurgery in our study. However, the main objective of endoscopic management is to treat obstructive hydrocephalus and re-establish quasi-physiological intraventricular CSF flux. Thus, the endoscopic clearance of the intraventricular blood and decompression of obstructive hydrocephalus may represent an option in these cases, even if the whole clot may not be suitable for endoscopic removal.

Clearing the third ventricle has been shown to dramatically improve CSF circulation and improve symptoms even in the absence of EVD [17]. Horvath and colleagues demonstrated that an endoscopic removal of the clot and third ventriculostomy offers a more adequate treatment option than external ventricular drainage in patients with primary IVH and hydrocephalus [19]. A ventriculostomy helps in physiological clearance of clots even in cases of incomplete evacuation [19]. In all our cases, we performed a third ventriculostomy and inserted an EVD for three days. This practice was reflected in the resolution of hydrocephalus in all cases except one. However, one has to be cautious while performing a third ventriculostomy in infratentorial lesions, which might be very difficult and even dangerous, especially in cases with large parenchymal haematoma. Thus, careful case selection is crucial. For that reason, infratentorial lesion patients included in our study had minimal parenchymal blood and mainly intraventricular blood, that could be aspirated and the third ventriculostomy easily accomplished.

Neuroendoscopy may offer some advantages over more traditional surgical approaches such as being less invasive than craniotomy. Endoscopic surgery however has its limitations. It is a technically demanding procedure. With increasing experience, our surgical time decreased from three hours to as short as 45 minutes. One also has to be careful during movements within the ventricles as this can cause cortical and subcortical damage, especially when using the rigid endoscopes. Navigation to the fourth ventricle and temporal horn cannot be achieved. Therefore, we used an infant feeding tube inserted through the aqueduct of Sylvius to clear haematoma from the posterior fossa and from atrium to the temporal horn. These problems can be addressed by using a flexible endoscope [17].

## Conclusion

Our study has shown encouraging results for the endoscopic management of hypertensive intraventricular haemorrhage with obstructive hydrocephalus. Patients with a GCS ≥ 9 on initial presentation demonstrate a better outcome with this minimally invasive method. Future improvements in instrumentation and surgical techniques, in particular flexible instrumentation, with appropriate case selection may help increase acceptability of this method and improve outcome.

## List of abbreviations

CSF: Cerebrospinal fluid

EVD: External ventricular drain

GCS: Glasgow Coma Scale

ICH: Intracerebral haemorrhage

IVH: Intraventricular haemorrhage

## Competing interests

The author(s) declare that they have no competing interests.

## Authors' contributions

YRY was the Chief Neurosurgeon, performed all the surgeries, conceived the study and participated in its design, coordination, interpretation and completion of the manuscript. GM and RS helped conceive the study, with interpretation of the data, and writing the manuscript. AB, GJ and AN were part of the surgical team and were responsible for the patient care and follow-up. All authors read and approved the manuscript.

## Pre-publication history

The pre-publication history for this paper can be accessed here:



## Figures and Tables

**Table 1:** Demographic and clinical characteristics of study population

**Parameter**		**Value % (n = 25)**
**Age**	41–50 yrs	16
	51–60 yrs	48
	61–70 yrs	32
	71–80 yrs	4
**Gender**	Male	68
	Female	32
**Site of bleed**	Putamen	44
	Thalamus	48
	Posterior fossa	8
**Haematoma volume**	30 – 40 ml	9
	41 – 50 ml	7
	51 – 60 ml	9
**Ventricular to Hemispheric ratio**	35 – 47 %	21
	> 47 %	4
**Glasgow Coma Score**	3–8	36
	9–12	64

**Table 2:** Summary of results

		**Pre-op GCS 3–8 (n = 9), %**	**Pre-op GCS 9–12 (n = 16), %**
**Resolution of hydrocephalus (CT scan)**		100	93.8
**Final Clinical Outcome (Glasgow Outcome Scale) at 6 months**	**Death**	55.5	6.3
	**Vegetative state**	-	-
	**Severe disability**	22.2	6.3
	**Moderate disability**	22.2	6.3
	**Good recovery**	0	81.3
